# The Current Status of Uptake of European BSS Directive (2013/59/Euratom) Requirements – Results of a Pilot Survey in European Radiology Departments with a Focus on Clinical Audit

**DOI:** 10.1186/s13244-019-0734-6

**Published:** 2019-05-09

**Authors:** European Society of Radiology (ESR), David C. Howlett, Adrian P. Brady, Guy Frija, Steve Ebdon-Jackson

**Affiliations:** Vienna, Austria

**Keywords:** Clinical audit, Radiation protection, Basic Safety Standards Directive (BSSD), 2013/59/Euratom, Radiology, Clinical governance

## Abstract

The recently implemented European Council Basic Safety and Standards Directive (BSSD), 2013/59/Euratom lays down core radiation protection standards for European radiology departments, with a mandatory requirement for supporting clinical audit processes.

A survey on behalf of the European Society of Radiology (ESR) was undertaken in November 2018 involving the ESR EuroSafe Imaging Star department network to assess compliance with selected key BSSD requirements, with emphasis also on clinical audit/re-audit.

64% of invited departments participated and the survey results revealed a lack of compliance with BSSD requirements even when allowing for work in progress within departments. Justification processes showed the lowest rates of compliance overall, with varying results relating to dose limits, patient information and significant accidental exposure notification. Questions around implementation of diagnostic reference levels (DRLs) revealed generally far higher compliance with requirements.

The survey findings confirm a lack of compliance with key BSSD radiation protection indicators and also a lack of supporting clinical audit structures. These findings are likely to be representative of the wider radiological community in Europe. There is a need for a co-ordinated response, involving relevant European agencies, national bodies and societies and also individual radiology departments to address these issues. ESR publications on clinical audit (Esperanto) and the 2018 EuroSafe Imaging Call for Action will be important components of this response.

**Electronic supplementary material:**

The online version of this article (10.1186/s13244-019-0734-6) contains supplementary material, which is available to authorized users.

## Key Points

- The Basic Safety and Standards Directive (BSSD), 2013/59/Euratom lays down standards for radiation protection that are legally required within EU member states.
- Clinical audit is a mandatory component of the BSSD; radiology departments will need to show evidence of compliance with the BSSD and supporting processes of clinical audit.
- A survey on behalf of the ESR was undertaken in 2018 amongst all radiology departments in the EuroSafe Imaging Star network, evaluating BSSD compliance.
- 64% of invited departments participated. Survey results demonstrated a generalised lack of compliance with BSSD radiation protection and clinical audit requirements.
- The survey results indicate the need for co-ordinated action, involving relevant European organisations and national bodies, to improve BSSD uptake and to develop clinical audit programmes within European radiology departments.

## Introduction

The European Basic Safety Standards Directive (BSSD) 2013/59/Euratom [1], laying down requirements for protection from the dangers associated with medical ionising radiation exposure, was adopted by the Council of the European Union (EU) in 2013, for transposition into the national legislation of EU Member States by February 2018. The BSSD directly impacts on all radiology departments and has been updated in light of new scientific evidence and guidelines. These changes from previous legislation are highlighted and summarised in a recent ESR publication [2]. Key aspects of the BSSD relevant to radiology departments include:

- Changes in justification processes and patient information requirements.
- Use of diagnostic reference levels (DRLs), and more stringent requirements around recording and reporting doses arising from radiological procedures.
- New dose limits for the eye and for occupational and student exposure.
- Clarification of the role of the medical physics expert.

Importantly, the BSSD specifically highlights the importance of clinical audit in radiation protection; carrying out clinical audit “in accordance with national procedures” is mandatory and a legal requirement as a result of 2018 BSSD transposition [1]. Alongside BSSD transposition, EuroSafe Imaging, as part of the ESR campaign to strengthen medical radiation protection across Europe, announced its second Call for Action in 2018 [3]. This publication contains 13 key actions around radiation protection associated with medical exposure, broadly highlighting and supporting the BSSD requirements [3]. Action 10 relates to strengthening the EuroSafe Imaging Stars network of radiology departments, embodying best practice in radiation protection.

This paper describes the results of a survey, distributed to all EuroSafe Imaging Star departments in late 2018. The survey examined departmental implementation of key, selected BSSD radiation protection requirements, also evaluating supporting clinical audit/re-audit infrastructure and processes. The results and their importance are discussed and recommendations made. The survey results and conclusions are best read in combination with a second survey undertaken at the same time, this survey examining the status of clinical audit and available infrastructure amongst European National Radiology Societies [4].

## Materials and Methods

A survey was prepared by members of the ESR Audit and Standards Subcommittee, the ESR EuroSafe Imaging Steering Committee and the ESR Office. The questionnaire was created using Survey Monkey, allowing ease of distribution, completion, return and analysis of results.

The questionnaire contained a range of questions covering selected key aspects of radiation protection as defined within the BSSD. For the majority of questions there were 3 components:

i) Has the requirement in question been implemented in the department?
ii) Does the department have a programme in place to audit the requirement?
iii) Is regular re-audit carried out (or planned)?

Questions were created to reflect core BSSD requirements, as also outlined within the EuroSafe Imaging 2018 Call for Action. The questions are included in (Additional file 1) Tables 2, 3.

The ESR has a well-established network of radiology departments within the EuroSafe Imaging Star network. These departments will have met the necessary requirements, including those relating to radiation protection (based upon the EuroSafe Imaging 2018 Call for Action), to allow recognition at the time of application or renewal of application based on self-assessment and submission of supporting evidence. Many of these departments will already engage in internal clinical audit; however it is important to note that a proportion of departments are likely to have acquired EuroSafe Imaging Star status prior to the BSSD and its transposition (date of acquisition was not formally assessed in the survey), so potentially there is likely to be some variation in implementation of BSSD requirements and related audit activity. It is also important to note that a proportion of EuroSafe Imaging Star departments are outside of the EU and therefore not legally bound by the BSSD; their results are however included.

The survey was launched to all EuroSafe Imaging Star radiology departments on the ESR database at the beginning of November 2018. An initial closing date for data submission was proposed for the end of that month, with a 2-week extension provided; the survey concluded on 14 December 2018. As part of the process, the anonymity of participants and their responses was guaranteed.

## Results

By the time of survey closure, responses to the questionnaire had been received from 66 out of 103 departments (64% response rate); 51 of these departments were from within the EU^1^.

Table 1 includes all those countries who have ≥ 1 EuroSafe Imaging Star department and also the number of departments in that country that responded. Note two countries (Switzerland and Turkey) are non-EU members but have EuroSafe Imaging Star departments and are included in the survey.

**Table 1:** Pilot Survey on uptake of European BSS Directive (2013/59 Euratom) requirements with particular focus on clinical audit in European radiology departments

Country	Responding EuroSafe Star Departments by country	Total no. of EuroSafe Imaging Star Departments by country
Austria	1	1
Belgium	2	2
Bulgaria	1	1
Croatia	1	1
Czech Republic	0	1
Finland	0	1
France	3	3
Germany	2	4
Greece	2	4
Hungary	8	8
Ireland	3	6
Italy	8	14
Lithuania	1	1
Poland	5	7
Portugal	7	7
Romania	1	1
Spain	3	3
Sweden	1	1
Switzerland	13	22
The Netherlands	2	2
Turkey	2	13
	66	103

The results of the survey (alongside the questions), are covered in (Additional file 1) Tables 2, 3 (examining written processes around accidental radiation exposure) and Table 3 (DRL based questions) are separated for ease of review.

**Table 2:** Pilot Survey on uptake of European BSS Directive (2013/59 Euratom) requirements with particular focus on clinical audit in European radiology departments

Question	A written information process is available for the referrer				
Written process informing key individuals/agencies of the exposure and subsequent analysis in the event of a significant accidental radiation exposure					
	Yes	No	Sometimes	Answered	Skipped
	26 (50.00%)	13 (25.00%)	13 (25.00%)	52	14
	the practitioner				
	Yes	No	Sometimes	Answered	Skipped
	43 (84.31%)	7 (13.73%)	1 (1.96%)	51	15
	the patient (or their representative)				
	Yes	No	Sometimes	Answered	Skipped
	35 (64.81%)	11 (20.37%)	8 (14.81%)	54	12
	the radiation protection competent authority				
	Yes	No	Sometimes	Answered	Skipped
	45 (76.27%)	11 (18.64%)	3 (5.08%)	59	7
	Does your department have a programme in place to audit this requirement?				
	Yes	No	In development	Answered	Skipped
	46 (82.14%)	8 (14.29%)	2 (3.57%)	56	10
	Is a regular re-audit carried out or planned?				
	Yes	No	In development	Answered	Skipped
	45 (80.36%)	7 (12.50%)	4 (7.14%)	56	10

**Table 3:** Pilot Survey on uptake of European BSS Directive (2013/59 Euratom) requirements with particular focus on clinical audit in European radiology departments

DRLs - Diagnostic Reference Levels	Has this requirement been implemented in your department?						Does your department have a programme in place to audit this requirement?						Is a regular re-audit carried out or planned?				
	Yes	No	In development	N/A (no DRLs in place or planned)	Answered	Skipped	Yes	No	In development	N/A (no DRLs in place or planned)	Answered	Skipped	Yes	No	In development	Answered	Skipped
DRLs established for typical radiodiagnostic examinations (X-ray)	49 (81.67%)	8 (13.33%)	3 (5.00%)		60	6	45 (88.24%)	4 (7.84%)	2 (3.92%)		51	15	45 (88.24%)	4 (7.84%)	2 (3.92%)	51	15
DRLs established for high-dose radiodiagnostic examinations (interventional radiology/CT)	57 (95.00%)	1 (1.67%)	2 (3.33%)		60	6	55 (93.22%)	3 (5.08%)	1 (1.69%)		59	7	54 (91.53%)	3 (5.08%)	2 (3.39%)	59	7
Consistency of DRLs in use in your department with current European recommendations (where available, note recent publication of paediatric DRLs)	49 (81.67%)	3 (5.00%)	7 (11.67%)	1 (1.67%)	60	6	49 (90.74%)	2 (3.70%)	3 (5.56%)	0	54	12	47 (87.04%)	3 (5.56%)	4 (7.41%)	54	12
Regular review of DRLs	54 (90.00%)	1 (1.67%)	4 (6.67%)	1 (1.67%)	60	6	48 (82.76%)	4 (6.90%)	6 (10.34%)	0	58	8	48 (82.76%)	4 (6.90%)	6 (10.34%)	58	8
Procedures for local review where DRLs consistently exceed recommendations and where corrective action is taken	51 (85.00%)	1 (1.67%)	8 (13.33%)		60	6	48 (82.76%)	3 (5.17%)	7 (12.07%)	0	58	8	47 (81.03%)	5 (8.62%)	6 (10.34%)	58	8

Key components of the results are also discussed in more detail in the discussion section.

## Discussion

The BSSD, 2013/59/Euratom, is a fundamentally important piece of legislation, one that will have both immediate and far reaching effects on all radiology departments across Europe. Implementation of the Directive will be underpinned by a process of external inspection, with European Union Member States determining how the BSSD requirements are met via national legislation. As previously mentioned, undertaking clinical audit “in accordance with national processes” is mandated within the BSSD; more specific reference is made to the clinical audit processes around radiation protection in the document R.P. No. 159, European Guidelines on Clinical Audit for Medical Radiological Practices [5]. The inferences are clear: as of February 2018 when the BSSD became a legal requirement, European Union Member States and their constituent radiology departments are required to comply with the BSSD, to have developed a supporting clinical audit programme, with the ability also to evidence compliance and relevant audit processes at the time of potential inspection by an external agency.

The BSSD uptake survey undertaken on behalf of the ESR has been initially directed to the ESR EuroSafe Imaging Star network. The survey response rate was good (64%) making significant response bias unlikely, and the co-operation of a large number of departments in completing survey returns is gratefully acknowledged. EuroSafe Imaging Star departments, by virtue of their voluntary involvement in the EuroSafe Imaging initiative, are likely to be aware of what constitutes good radiation protection practice. Many are also likely to have implemented internal clinical audit programmes as part of the EuroSafe Imaging Stars self-assessment process. It is not unreasonable to conclude that this group of departments, with a geographic distribution across Europe (and beyond), is likely to be at the forefront of adoption of procedures/practices allowing BSSD compliance.

The survey, covering implementation of core BSSD requirements and development of supporting clinical audit processes (importantly also including re-audit, to close the audit loop and to re-assess parameters where continual monitoring is required), was distributed as a pilot survey. No standards/targets were included which would have moved the emphasis towards an audit. It is important to note however, that all the BSSD requirements covered in the survey (and the need to audit/re-audit), if considered as standards for an audit, would be fixed, compulsory and with a target of 100% (by virtue of their derivation from the BSSD).

Some observations can be made on evaluation of the survey results:

- A compliance figure of 100% was only achieved for one parameter (the ability of CT equipment to record patient dose). 100% compliance was not recorded for supporting clinical audit/re-audit in any area, even allowing for work in progress.
- 82% of departments had an existing process of clinical audit in place to evaluate local radiation practice (as per BSSD).
- In terms of what was done best, the questions surrounding DRLs generally demonstrated the highest levels of compliance and supporting audit development.
- As a group, the questions surrounding justification processes, a key component of optimisation, (delegation, transferance, training documentation) revealed relatively poor levels of compliance with BSSD procedural and audit requirements.
- Questions around dose limits for workers (including breast-feeding/pregnancy and students/apprentices) demonstrated mixed levels of positive responses for policy implementation and supporting audit. Some very low levels of compliance were notable (28.6% dose limit eye, 29% dose limit skin for students and apprentices), in other areas (dose limits breast feeding/pregnancy) far higher levels were apparent.
- Responses confirmed generally good medical physics expert availability (and supporting audit of this activity).
- Patient information and significant accidental radiation exposure written mechanisms also showed variable levels of compliance. 77% of departments had a pre-procedural patient information mechanism of some kind in place. Only 50% of departments had a written information process in place to inform the referrer in cases of accidental radiation exposure, with 76% fulfilling their obligation by informing the radiation protection competent authority.

One has to be cautious when trying to draw conclusions from survey data, in terms of accuracy and generalisability. It is also important to note that in many departments work is in progress to improve BSSD implementation, including clinical audit development – so results are likely to improve. There was a good survey response rate of 64% from a group of radiology departments who are likely to be motivated and cognisant of BSSD requirements. The survey results do indicate that there is currently a lack of overall compliance with the requirements of the BSSD. This has been observed in EuroSafe Imaging Star departments but is likely to be representative of wider European radiology department current practice. There is a pressing need to further highlight the importance of the BSSD across Europe and for relevant European Agencies, national governmental bodies and societies to collaborate and promote active uptake of the BSSD within all Member States and their radiology departments. The ESR will have a central role in this process, working with the European Commission, the Heads of the European Radiological Protection Competent Authorities (HERCA) and Member State National Radiology Societies. The ESR has produced Esperanto – a Guide to Clinical Audit and Clinical Audit Tool. First published in 2017, this guide is designed to support radiology departments in developing a clinical audit programme, and contains numerous radiation protection audit templates [6]. An enhanced version of Esperanto is to be launched at the European Congress of Radiology (ECR) in 2019 [7], containing an expanded audit guide with an additional focus on the legal requirements around audit, and an increase in audit templates (30 in total, covering both radiation protection and also clinical practice and service provision). The EuroSafe Imaging Call for Action, 2018 [3] is the ESR flagship campaign promoting and strengthening quality and safety in medical imaging. Radiation protection and clinical audit have increasingly high profiles in European radiology meetings (including ECR) and publications (for example the recent European Commission publication, European Guidelines on Diagnostic Reference Levels For Paediatric Imaging [8]). An essential part of addressing issues around BSSD compliance is likely to include establishing/facilitating functional national inter-departmental audit and clinical best practice networks via National Radiology Societies, working with the ESR and other specialist bodies. This area is covered in more detail in the accompanying National Radiology Societies Clinical Audit survey [4].

## Conclusion

This survey of EuroSafe Imaging Star departments has revealed a lack of compliance with many BSSD radiation protection requirements and supporting clinical audit processes. These findings are likely to be representative of current practice in radiology departments across Europe and indicate the need for further action. Co-operation between relevant European organisations (including the ESR), national bodies and governmental institutions is required to embed a co-ordinated and structured radiation protection and supporting clinical audit programme across European radiology departments, to meet the requirements of the BSSD and to enhance patient experience, safety and outcomes.

## Additional file

Additional file 1: Pilot Survey on uptake of European BSS Directive (2013/59 Euratom) requirements with particular focus on clinical audit in European radiology departments. (XLSX 17 kb)

## Footnotes

1 ESR National Radiological Societies of European countries as defined by the ESR Executive Council are listed at the following link: https://www.myESR.org/about/organisation/institutional-member-societies. At the time of the survey there were 47 ESR national member societies. Subsequent to the survey, a further society became ESR member. As of February 2019, the ESR counts 48 national member societies.
